# Evaluation of a Dedicated Software “Elements™ Spine SRS, Brainlab^®^” for Target Volume Definition in the Treatment of Spinal Bone Metastases With Stereotactic Body Radiotherapy

**DOI:** 10.3389/fonc.2022.827195

**Published:** 2022-05-12

**Authors:** Maximilien Rogé, Ahmed Hadj Henni, Yasmine Adda Neggaz, Romain Mallet, Chantal Hanzen, Bernard Dubray, Elyse Colard, David Gensanne, Sébastien Thureau

**Affiliations:** ^1^Départment of Radiation Oncology, Centre Henri Becquerel, Rouen, France; ^2^QuantIF-LITIS EA4108, University of Rouen, Rouen, France

**Keywords:** stereotactic body radiotherapy, clinical target volume, spinal metastases, software, artificial intelligence (AI)

## Abstract

**Introduction:**

Stereotactic body radiotherapy (SBRT) is a treatment option for spine metastases. The International Spine Radiosurgery Consortium (ISRC) has published consensus guidelines for target delineation in spine SBRT. A new software called Elements™ Spine SRS by Brainlab^®^ that includes the module Elements SmartBrush Spine (v3.0, Munich, Germany) has been developed specifically for SBRT treatment of spine metastases, and the latter provides the ability to perform semiautomatic clinical target volume (CTV) generation based on gross tumor volume (GTV) localization and guidelines. The aims of our study were to evaluate this software by studying differences in volumes between semiautomatic CTV contours compared to manual contouring performed by an expert radiation oncologist and to determine the dosimetric impact of these differences on treatment plans.

**Methods:**

A total of 35 volumes (“Expert GTV” and “Expert CTV”) from 30 patients were defined by a single expert. A semiautomatic definition of these 35 CTVs based on the location of “Expert GTV” and following ISRC guidelines was also performed in Elements SmartBrush Spine (“Brainlab CTV”). The spatial overlap between “Brainlab” and “Expert” CTVs was calculated using the Dice similarity coefficient (DSC). We considered a threshold of 0.80 or above to indicate that Elements SmartBrush Spine performed very well with adequate contours for clinical use. Two dosimetric treatment plans, each corresponding to a specific planning target volume (PTV; Expert PTV, Brainlab PTV), were created for 11 patients.

**Results:**

We showed that “Brainlab CTV” and “Expert CTV” mean volumes were 29.8 ± 16.1 and 28.7 ± 15.7 cm^3^, respectively (p = 0.23). We also showed that the mean DSC for semiautomatic contouring relative to expert manual contouring was 0.85 ± 0.08 and less than 0.80 in five cases. For metastases involving the vertebral body only (n = 13,37%), the mean DSC was 0.90 ± 0.03, and for ones involving other or several vertebral regions (n = 22.63%), the mean DSC was 0.81 ± 0.08 (p < 0.001). The comparison of dosimetric treatment plans was performed for equivalent PTV coverage. There were no differences between doses received by organs at risk (spinal cord and esophagus) for Expert and Brainlab PTVs, respectively.

**Conclusion:**

The results showed that the semiautomatic method had quite good accuracy and can be used in clinical routine even for complex lesions.

## Introduction

Spine metastases are a common manifestation of cancer and can be associated with “skeletal-related events” (SRE) that are associated with, for example, back pain, fracture, hypercalcemia, or medullar compression.

Conformal, palliative external beam radiotherapy (cEBRT) is a treatment modality for spine metastases that can be useful in certain situations such as for pain control, bone consolidation, or postoperative cases (1–3). Fractionation regimens commonly used for cEBRT are 8 Gy in 1 fraction, 20 Gy in 5 fractions, and 30 Gy in 10 fractions.

Stereotactic body radiotherapy (SBRT) is another treatment option for spine metastasis which delivers high-dose radiation with millimetric precision and with steep dose gradients between target volumes and adjacent organs at risk (OARs) such as the spinal cord or esophagus.

Dose and fractionation schemes are heterogeneous between different centers and studies, but frequent protocols used are 16–24 Gy in 1 fraction, 24 Gy in 2 fractions, 24–27 Gy in 3 fractions, and 30–35 Gy in 5 fractions (4).

A high biologically equivalent dose (BED) provides good local control. Indeed, multiple prospective and retrospective studies show local control rates of about 80%–90% at 1 year (5).

SBRT for spine metastases appears to be an interesting option for oligometastatic patients in order to increase overall survival (6–8).

To compare results of control rates or survival between different doses and fractionation schemes, the definition of target volumes should be standardized first.

The International Spine Radiosurgery Consortium (ISRC) has published consensus guidelines for target delineation in spine SBRT based on expert opinions for 10 representative cases (9). The clinical target volume (CTV) should include areas of potential microscopic extension. For example, if the metastasis has invaded the vertebral body and pedicle, the entire involved region (vertebral body and pedicle) and the adjacent region (transverse process and lamina) should be included in the CTV.

A new software called Elements Spine SRS by Brainlab^®^ (v3.0, Munich, Germany) was developed specifically for treatment of spine metastases using SBRT and offers clinical efficiency for both treatment planning and delivery, where the feasibility of clinical application of this software has already been evaluated in a previous study (10).

In the Elements Spine SRS workflow, the Elements SmartBrush Spine module can perform semiautomatic CTV generation based on the gross tumor volume (GTV) location, following the ISRC consortium guidelines.

The aims of our study were to evaluate this module by studying differences in target volumes between semiautomatic CTV contours compared to manual contouring performed by an expert radiation oncologist and to determine the dosimetric impact of these differences on treatment plans.

## Materials and Methods

This retrospective monocentric study was approved by the local ethics committees and registered at www.health-data-hub.fr.

### Patient Population

The inclusion criteria were as follows: treatment between January 2018 and February 2021 at our department of radiation oncology for one or several spinal metastases treated by SBRT.

The exclusion criteria were as follows: postoperative SBRT or spinal metastasis involving soft tissues, since only preoperative consortium guidelines are implemented in the current 3.0 version of the software module (Elements SmartBrush Spine).

For patients who had several spinal metastases, each involved and treated vertebral level or lesion was defined and analyzed as a separate metastasis. Each vertebral lesion had its own set of contours and was reported independently with respect to results.

### Expert Target Volume Definition

Expert target volumes were defined with a standard contouring platform (Eclipse 15.6, Varian Medical Systems, Palo Alto, CA, USA) by a single radiation oncologist with solid expertise and experience in spinal metastasis SBRT. In our study, manual segmentation was considered the gold standard and served as the ground truth for the semiautomatic method.

The GTV corresponded to the tumor volume visible on the simulation CT, taking the information provided by other imaging modalities (macroscopic enhancing lesion on co-registered MRI or pathological uptake on PET CT) into account. This GTV was called “Expert GTV”.

The CTV included the GTV, abnormal marrow signals suspicious for microscopic invasion, and adjacent normal bony expansion to take into account subclinical spread. The CTV was created according to international guidelines (9). This CTV was called “Expert CTV”.

The PTV was defined as the CTV with margins to account for uncertainties in beam alignment, variation in patient position, organ motion, and other uncertainties. A margin of 2 mm was applied to the CTV to obtain the PTV except close to the spinal cord, where the margin was reduced to 0 mm. We used this methodology to create the “Expert PTV” based on the “Expert CTV”.

The expert radiation oncologist also manually defined the esophagus and spinal cord on the simulation CT, taking into account the information provided by the co-registered MRI.

### Brainlab Target Volume Definition

Planning CT scan, co-registered MRI volumetric 3D sequences (T1, T2, and post-contrast T1), “Expert GTV,” and OAR contours were imported in Elements Spine SRS.

For each spinal metastasis, we proceeded with the different steps as follows:

(A) “Elements Image Fusion” and “Elements Curvature Correction Spine”: software modules for fusion between the planning CT and MRI sequences where individual rigid image co-registrations were calculated for each vertebra and, thereafter, a single 3D deformation field that matches all vertebrae in the fused images at the same time was determined (11).

(B) “Elements SmartBrush Spine”: first allowed a recognition of imported “Expert GTV”. Then, the clinical target volume was automatically generated by the software based on the location of the “Expert GTV” following the IRSC guidelines. This semiautomatic CTV was called “Brainlab CTV.” Because of the necessity to manually define the GTV beforehand, we use the term semiautomatic CTV and not the term automatic CTV.

(C) “Elements Object Manipulation”: using the same methodology as we did for “Expert PTV”, we created “Brainlab PTV” based on “Brainlab CTV”.

### Dosimetric Study

Two dosimetric treatment plans, each corresponding to a specific target volume (“Expert PTV” and “Brainlab PTV”), were created for each of the patients included in this section (11 patients) with a prescribed dose of 35 Gy in 5 fractions of 7 Gy each.

The objective of achieving identical PTV coverage for the dosimetric study was based on three dosimetric indices: Paddick conformity index (PCI), homogeneity index (HI), and gradient index (GI).

For equivalent PTV coverage, the comparison was made on the doses delivered to OARs.

All treatment plans were calculated with volumetric modulated arc therapy (VMAT) according to our dosimetric protocol using three coplanar arcs for 6-MV photons at 600 MU/min and collimator angles of 45°, 315°, and 95°. All treatment plans were calculated using the same planning system Elements Spine SRS (Elements v3.0, Monte Carlo algorithm, 2 mm grid).

### Outcomes and Statistical Methods

The performance of the Elements SmartBrush Spine module (“Brainlab CTV”) was compared to manual contouring (“Expert CTV”) for 30 patients.

The spatial overlap between “Expert CTV” and “Brainlab CTV” was calculated using the Dice similarity coefficient (DSC) which ranges between 0 and 1 (0 = no overlap, 1 = total congruence) (12).

The DSC was calculated according to:


$$DSC\;\left(Expert\;CTV,\;Brainlab\;CTV\right)=\frac{2\left(Expert\;CTV\;\cap \;Brainlab\;CTV\right)}{\left(Expert\;CTV\;\right)+\;\left(Brainlab\;CTV\right)}$$

We considered a DSC threshold of 0.80 and above to indicate that Elements SmartBrush Spine performed very well with adequate contours for clinical use.

The following three dosimetric indices were also analyzed:


$$Paddick\;conformity\;index:\;PCI=\frac{\left(PTV\;Volume\;\cap \;V95\%\right)}{PTV\;Volume}\times \frac{\left(PTV\;Volume\;\cap V95\%\right)}{V95\%}$$

$$Homogeneity\;index:\;HI=\frac{D2\%-D98\%}{D50\%}$$

$$Gradient\;index:\;GI=\frac{50\%\;isodose\;volume}{100\%\;isodose\;volume}$$

To determine the difference between the two CTVs (cm^3^) and the DSC based on the spinal metastasis location as well as the dosimetric study, Wilcoxon signed-rank tests with continuity correction were conducted. We carried out a linear regression with the DSC and the metastasis location. The results were considered significant for a p-value below 0.05. All statistical tests were performed using the statistical software R (version 4.1.0).

## Results

### Patient Characteristics

From January 2018 to February 2021, characteristics from 35 patients who presented with spinal metastases and underwent SBRT in our center were retrospectively analyzed. Following the inclusion criteria previously specified, 30 patients and 35 lesions were identified and included in the final analysis (flowchart available in the **Appendix**).

The median age at treatment was 63 (30–76) years, and the most common types of cancer were breast, lung, and prostate. Among the 35 lesions, 13 involved the vertebral body only and 22 involved other vertebral segments.

Treatment plans were created for a group of 11 patients (11 lesions) who were randomly selected among the 30 patients. This subgroup of 11 patients was found to be representative of the main group of 30 patients. **Table 1** summarizes patient and tumor characteristics.

**Table 1:** Patient and tumor characteristics.

All patients (n = 30)			Dosimetric study (n = 11/30, 37%)
Sex	Female	18 (60%)	4 (36%)
	Male	12 (40%)	7 (64%)
Median age (range)		63 years old (30–76)	62 years old (46–72)
Type of cancer	Breast	10 (33%)	4 (36%)
	Prostate	8 (27%)	5 (46%)
	Lung	8 (27%)	1 (9%)
	Other *^a^*	4 (13%)	1 (9%)
Tumor histology	Adenocarcinoma	17 (56%)	6 (55%)
	Ductal carcinoma	8 (27%)	3 (27%)
	Other *^a^*	5 (17%)	2 (18%)
Number of treated spinal metastases		35	11
Spinal metastases localization	Cervical	2 (6%)	1 (9%)
	Thoracic	21 (60%)	6 (55%)
	Lumbar	12 (34%)	4 (36%)
Anatomical site	Vertebral body only	13 (37%)	3 (27%)
	Vertebral body + other segment(s)	13 (37%)	4 (36%)
	Other segments (spinous process, lamina, transverse process, …)	9 (26%)	4 (36%)

a Details in the **Appendix**.

### Elements SmartBrush Spine Study

#### Volumes

**Figure 1:**
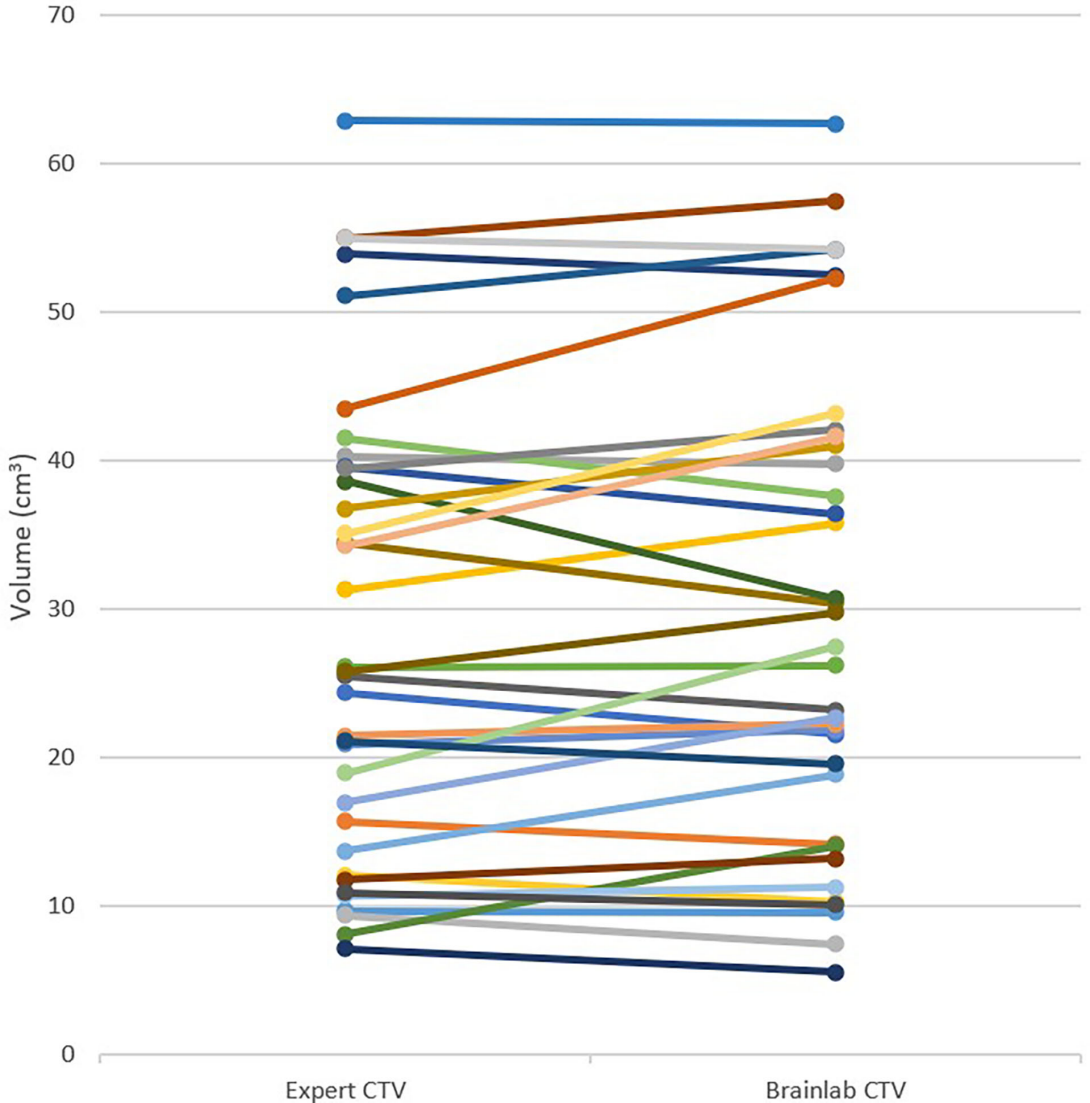
"Expert CTV" and "Brainlab CTV" volumes (Cm3) for each lesion.

The “Expert GTV” mean volume was 5.7 ± 6.5 cm^3^. The “Expert CTV” and “Brainlab CTV” mean volumes were 28.7 ± 15.7 cm^3^ and 29.8 ± 16.1 cm^3^, respectively. There was no difference found between the two CTVs for any of the lesions, including lesions that only involved the vertebral body. Nevertheless, we observed a non-significant difference (p = 0.06) between the two CTVs for lesions that involved other vertebral regions. **Table 2** reports the CTV measurements, and **Figure 1** depicts “Expert CTV” and “Brainlab CTV” volumes for each lesion.

**Table 2:** Clinical target volume (CTV) measurements for all lesions, for metastases which only involved vertebral body, and for other lesions.

	Mean “Expert CTV” (cm^3^) ± *standard deviation (SD)*	Mean “Brainlab CTV” (cm^3^) ± *SD*	*p value*
**All lesions (n = 35)**	28.7 ± *15.7*	29.8 ± *16.1*	*p = 0.23*
*Vertebral body (n = 13)*	32.8 ± *14.4*	32.1 ± *15.2*	*p = 0.54*
*Other lesions (n = 22)*	26.2 ± *16.2*	28.4 ± *16.8*	*p = 0.06*

#### Accuracy

The mean DSC for the semiautomatic method approach relative to the expert manual segmentation was 0.85 ± 0.08.

For metastases that involved the vertebral body only (n = 13, 37%), the mean DSC was 0.90 ± 0.03. For lesions that involved other vertebral regions (n = 22, 63%), the mean DSC was 0.81 ± 0.08. We observed a statistically significant difference between these two mean values (p < 0.001).

There was a statistically significant relationship between DSC and metastasis location. The DSC for metastases that involved the vertebral body only was on average 0.09, superior to the DSC for lesions involving other vertebral regions (p < 0.01).

**Figure 2:**
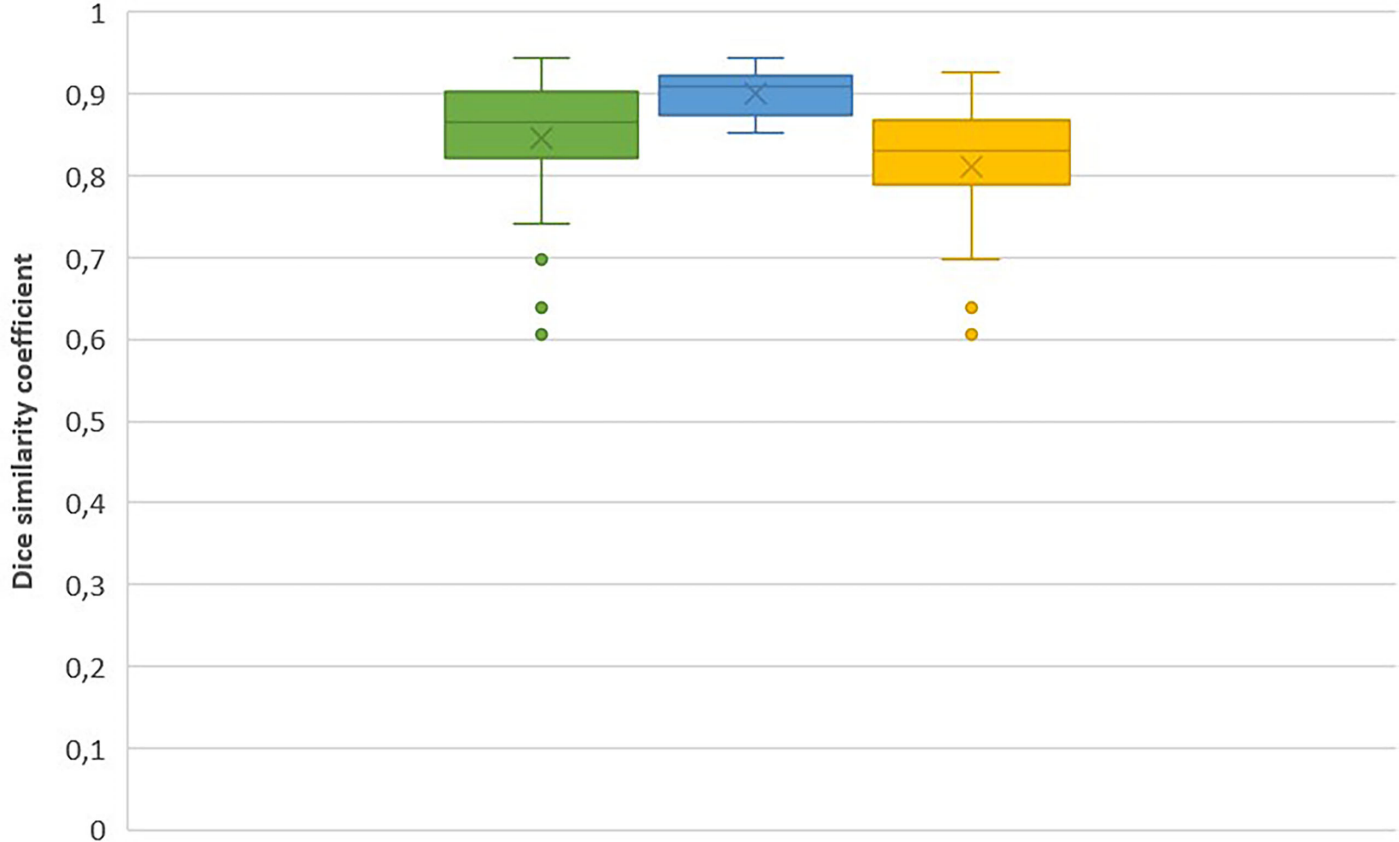
DCS for all lesions (green); for metastasis which only involved vertebral body (blue) and for all other lesions (yellow), respectively. Cross represents mean value.

Corresponding box plots of both DSC measures are shown in **Figure 2**. The DSC box plots show the highest values with low variability for lesions that only involved the vertebral body.

**Figure 3:**
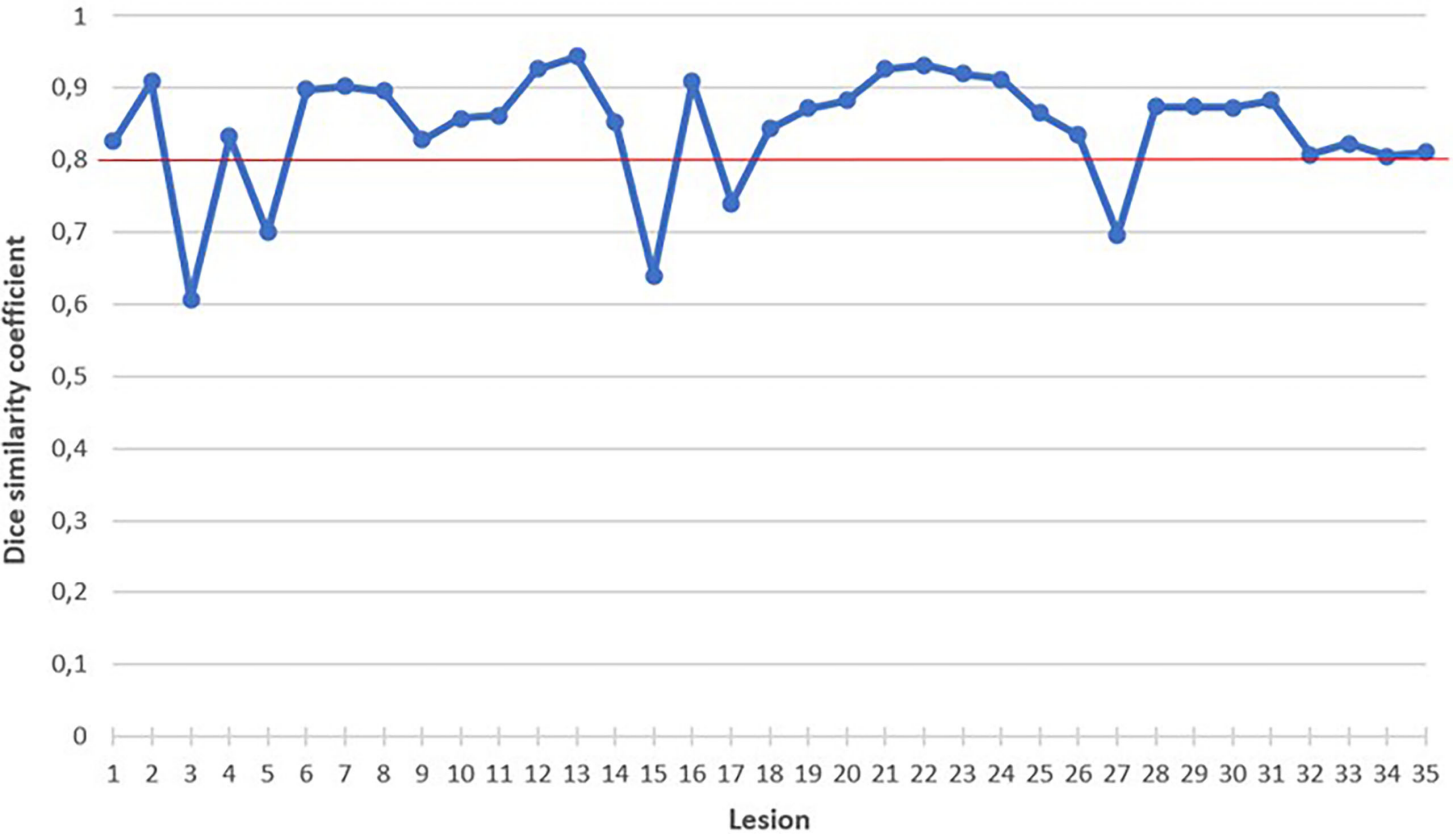
Dice similarity coefficient for each lesion. Red line represents our DSC threshold of 0.80. .

As shown in **Figure 3**, the semiautomatic method performed poorly in five cases with a DSC < 0.80. Two of these cases are illustrated in the **Appendix**.

For the 11 lesions included in the dosimetric study, the mean DSC was 0.84 ± 0.10.

### Dosimetric Study

“Brainlab PTV” and “Expert PTV” volumes did not differ significantly with 50.63 ± 22.65 cm^3^ and 49.17 ± 22.60 cm^3^ (p = 0.772), respectively. There was no difference found between “Brainlab” and “Expert” PTVs in terms of monitor Uuits (MU) with MU_BrainLab_ being 14,154 ± 2,169 and MU_Expert_ being 13,669 ± 2,666 (p = 0.863).

**Figure 4:**
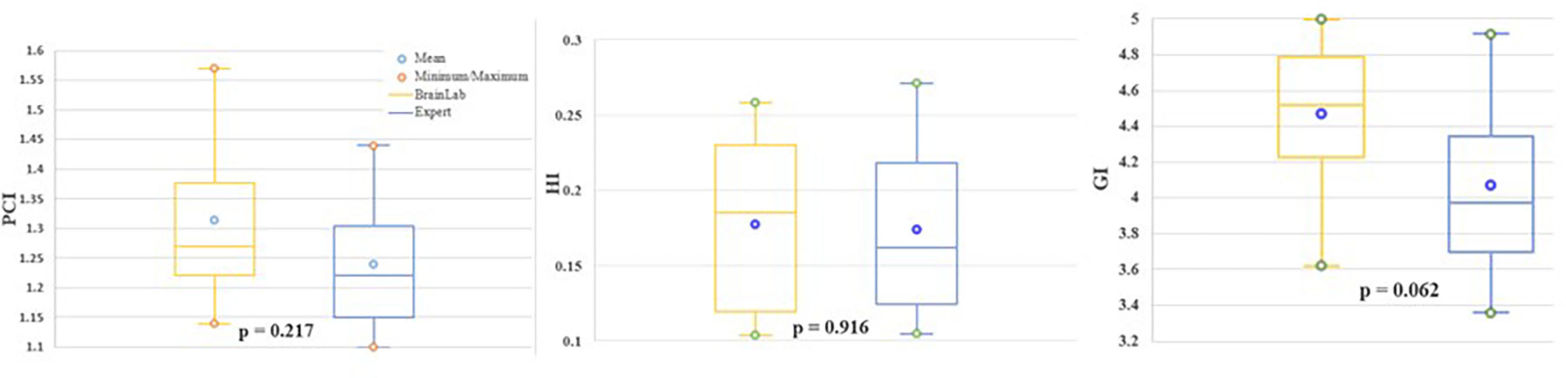
For evaluation of "Brainlab PTV (yellow) and "Expert PTV" (blue) coverage, Paddick corformity index (PCI, left), Homogeneity (HI, middle), and Gradient index (GI, right) were calculated.

Equivalent PTV coverage was obtained as shown with different dosimetric indices. There were no differences for the homogeneity index [HI_BrainLab_ = 0.18 ± 0.06 *versus* HI_Expert_ of 0.17 ± 0.06 (p = 0. 916)], Paddick conformity index [PCI_BrainLab_ = 1.33 ± 0.15 *versus* PCI_Expert_ = 1.24 ± 0.12 (p = 0.217)], and gradient index [GI_BrainLab_ = 4.47 ± 0.41 *versus* GI_Expert_ 4.07 ± 0.49 (p = 0.062)]. Results are summarized in **Figure 4**.

**Figure 5:**
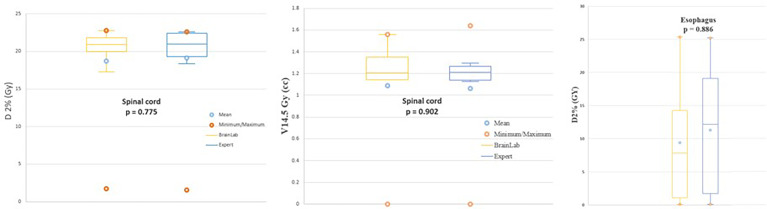
D2%(Gy) received by the spinal cord (left), volume (CM3) of spinal cord receiving 14.5 Gy (middle) and D2%(Gy) received by the esophagus (right) for treatment plans based on the "Brainlab PTV" (yellow) and "Expert PTV" (blue).

For equivalent coverages, the results for OARs are shown in **Figure 5**. For the spinal cord, considered to be the most critical OAR, doses received for the two treatment plans for “Brainlab PTV” and “Expert PTV” were equivalent for D_2%_ (Gy), 19.03 ± 6.27 and 19.10 ± 6.34 (p = 0.775), respectively. The mean V14.5 Gy (cm^3^) was also similar with 1.10 ± 0.43 cm^3^ and 1.06 ± 0.47 for “Brainlab PTV” and “Expert PTV” treatment plans, respectively (p = 0.902). D_2%_ (Gy) received by the esophagus was on average 11.69 ± 10.96 Gy for the “Brainlab PTV” treatment plans versus 11.30 ± 10.61 Gy for the “Expert PTV” treatment plans (p = 0.886).

## Discussion

Our study compared manual to semiautomatic contouring methods (based on Elements SmartBrush Spine SRS) for segmentation of CTVs for spinal bone metastases treated with SBRT. We investigated the quality of the semiautomatic method using manual segmentation as the gold standard. The DSC was used as a robust performance indicator, like in many other studies (11, 13, 14). In addition to the DSC, we also compared the volumes of the two CTVs (cm^3^).

“Expert CTV” and “Brainlab CTV” mean volumes were 28.7 ± 15.7 cm^3^ and 29.8 ± 16.1 cm^3^ (p = 0.23), respectively. We showed that the mean DSC was 0.85 ± 0.08 and less than 0.80 in five cases.

For metastases which exclusively involved the vertebral body (n = 13, 37%), the mean DSC was 0.90 ± 0.03 with no difference between the two CTVs, with “Expert CTV” and “Brainlab CTV” mean volumes measured at 32.8 ± 14.4 cm^3^ and 32.1 ± 15.2 cm^3^ (p = 0.54), respectively.

For metastases that involved other segments (n = 22, 63%), the mean DSC was 0.81 ± 0.08 with a non-significant difference between the two CTVs, with “Expert CTV” and “Brainlab CTV” mean volumes measured at 26.2 ± 16.2 cm^3^ and 28.4 ± 16.8 cm^3^ (p = 0.06), respectively.

There was a statistically significant relationship between DSC and metastasis location. The DSC for metastases that involved the vertebral body only was on average 0.09, superior to the DSC of lesions which involved other vertebral regions (p < 0.01). Indeed, for a metastasis invading only the vertebral body, there is less variability and difficulty in identifying the invaded vertebral segment and then determining the CTV (vertebral body and homolateral pedicle). In contrast, in the case of metastases invading other vertebral segments, the identification of the invaded vertebral segments and those to be included in the CTV may be more difficult (two cases illustrated in the **Appendix**).

Elements Spine SRS by Brainlab^®^ (Munich, Germany) was developed specifically for treatment of spine metastases with SBRT and has already been evaluated in clinical routine in a previous study by Giaj-Levra et al. (10). From April 2018 to April 2019, 54 spinal metastases in 32 patients were treated. With a median follow-up of 6 months (range 3–12), the local control rates at 6 and 9 months were 86% and 86%, respectively. The authors showed a control rate concordant with the results of other studies (5) and concluded that Elements Spine SRS is a feasible approach.

Two pilot feasibility studies have previously evaluated other modules of the software (i.e., “Elements Curvature Correction Spine” and “Elements Anatomical Mapping”) (11, 15).

To our knowledge, this is the first study that specifically evaluates the “Elements SmartBrush Spine” segmentation for CTVs. In addition to the two studies previously mentioned, our study has allowed us to validate the whole Elements Spine SRS workflow accurately.

Our study was conducted using a robust methodology: for all lesions, expert volumes (GTVs and CTVs) were contoured by a single radiation oncologist blinded to the contours already done for treatment delivery by another radiation oncologist. To improve the quantitative analysis, we also analyzed the CTV volumes (cm^3^).

To further increase the robustness of our work, we also undertook a dosimetric study for a subgroup that was representative of all our patients. The purpose of this dosimetric study was to compare the dose received by OARs when volumes are not perfectly congruent. We determined that the differences were not dosimetrically relevant.

However, our study has some limits. Effective time saving is one of the intended endpoints with automatic segmentation, but we did not evaluate time consumption for either method used in our study.

Delineation of target volumes is an important step for which inter- and intra-observer variability exists. Cox et al. (9) showed that for 10 contours done by experts, the mean GTV kappa agreement level was at 0.65 (0.54–0.79) and the mean CTV kappa agreement level was at 0.64 (0.54–0.82).

Giaj-Levra et al. evaluated in a recent study the advantage of the new automatic target contouring tool (Elements Spine SRS, Brainlab^®^) for the definition of GTV (16). Using data of 20 patients with spinal metastases outlined by three independent observers, they showed that the agreement of GTV contours outlined by independent observers was superior with the use of the automatic tool compared to manually outlined contours (mean Dice coefficient 0.75 vs. 0.57, p = 0.048).

We could have evaluated this variability to further improve the robustness of our gold standard method but also to increase the accuracy of “Brainlab CTV” based on the “Expert GTV” definition.

Furthermore, as already shown by Giaj-Levra concerning the GTV delineation, the use of an automatic target contouring tool could also minimize the inter- and intra-observer variability concerning the CTV delineation.

Lastly, because of our exclusion criteria (postoperative SBRT and spinal metastasis involving soft tissues), an “Elements SmartBrush Spine” evaluation for all clinical situations was not possible.

## Conclusion

Accurate and consistent delineation of the CTV is important for radiotherapy outcomes, especially for SBRT. The present study evaluated a solution for semiautomatic segmentation of CTVs for spine metastases treated with SBRT. The results showed that the semiautomatic method had quite good accuracy and can be used in clinical routine even for complex lesions. Future areas of study include evaluating intra- and interobserver reproducibility for expert volume definition (GTV and CTV) and time consumption using either method.

## Data Availability Statement

The original contributions presented in the study are included in the article/**Supplementary Material**. Further inquiries can be directed to the corresponding author.

## Author Contributions

MR, AH, ST, and DG contributed to the conception and design of the study. MR organized the database. MR and AH performed the statistical analysis. MR wrote the first draft of the manuscript. MR, AH, and YA wrote sections of the manuscript. All authors contributed to manuscript revision and read and approved the submitted version.

## Conflict of Interest

The authors declare that the research was conducted in the absence of any commercial or financial relationships that could be construed as a potential conflict of interest.

## Publisher’s Note

All claims expressed in this article are solely those of the authors and do not necessarily represent those of their affiliated organizations, or those of the publisher, the editors and the reviewers. Any product that may be evaluated in this article, or claim that may be made by its manufacturer, is not guaranteed or endorsed by the publisher.
