# Supplementary material for: Evaluation of a Dedicated Software “Elements™ Spine SRS, Brainlab®” for Target Volume Definition in the Treatment of Spinal Bone Metastases With Stereotactic Body Radiotherapy
Source: Front Oncol. 2022 May 12;12:827195. doi: 10.3389/fonc.2022.827195 (PMC9133331; doi:10.3389/fonc.2022.827195)

## Supplementary figure: Flowchart

##
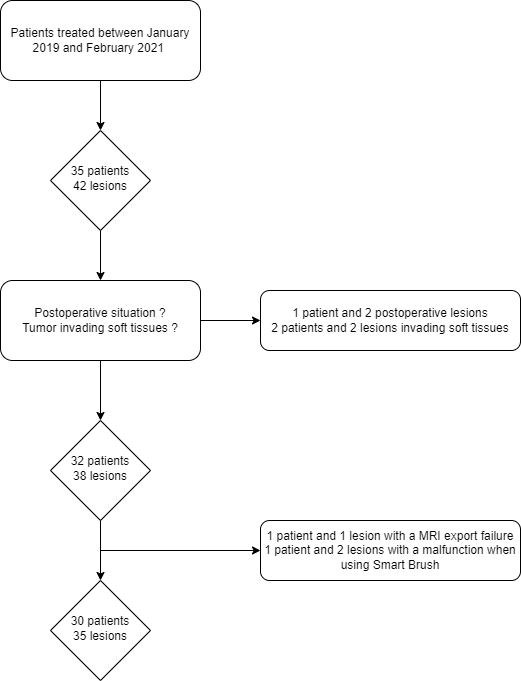


## Supplementary table: Details of types of cancer and histologies

| Type of cancer | Breast | 10 (33%) |
| --- | --- | --- |
|  | Prostate | 8 (27%) |
|  | Lung | 8 (27%) |
|  | Pancreas | 1 (3%) |
|  | Testicle | 1 (3%) |
|  | Bowel | 1 (3%) |
|  | Unknow | 1 (3%) |
| Tumor histology | Adenocarcinoma | 17 (57%) |
|  | Ductal carcinoma | 8 (27%) |
|  | Lobular carcinoma | 1 (3%) |
|  | Undifferentiated carcinoma | 1 (3%) |
|  | Seminoma | 1 (3%) |
|  | Neuroendocrine tumor | 2 (7%) |

**Supplementary figure**: T5 metastasis, first case

***The expert considered that the GTV (red) involved the vertebral body and right pedicle and didn’t invade the right transverse process or left pedicle. Therefore, “Expert CTV” (salmon, down right) included the vertebral body, right pedicle and left transvers process and lamina.
Elements Spine SRS considered that the GTV involved the vertebral body, left and right pedicles and right transverse process. Therefore, “Brainlab CTV” (pink, down left) included the whole vertebra.***


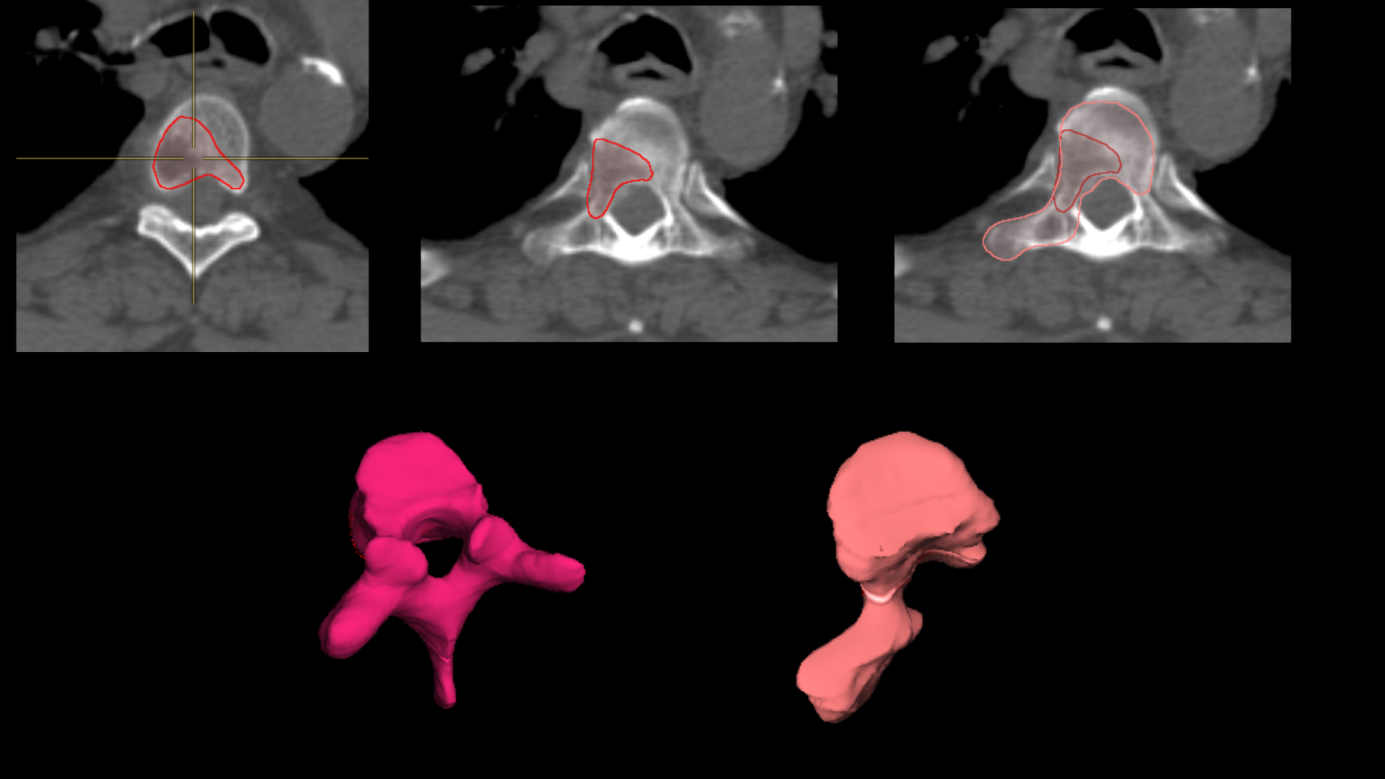


## Supplementary figure, T5 metastasis, second case

***The expert considered that the GTV (red) involved the vertebral body, right pedicle and didn’t invade the right transverse process or left pedicle. Therefore, “Expert CTV” (salmon, down right) included the vertebral body, right and left pedicles and left transvers process and lamina.
Elements Spine SRS considered that the GTV involved the vertebral body, left and right pedicles and the right transverse process. Therefore, “Brainlab CTV” (pink, down left) included the whole vertebra.***


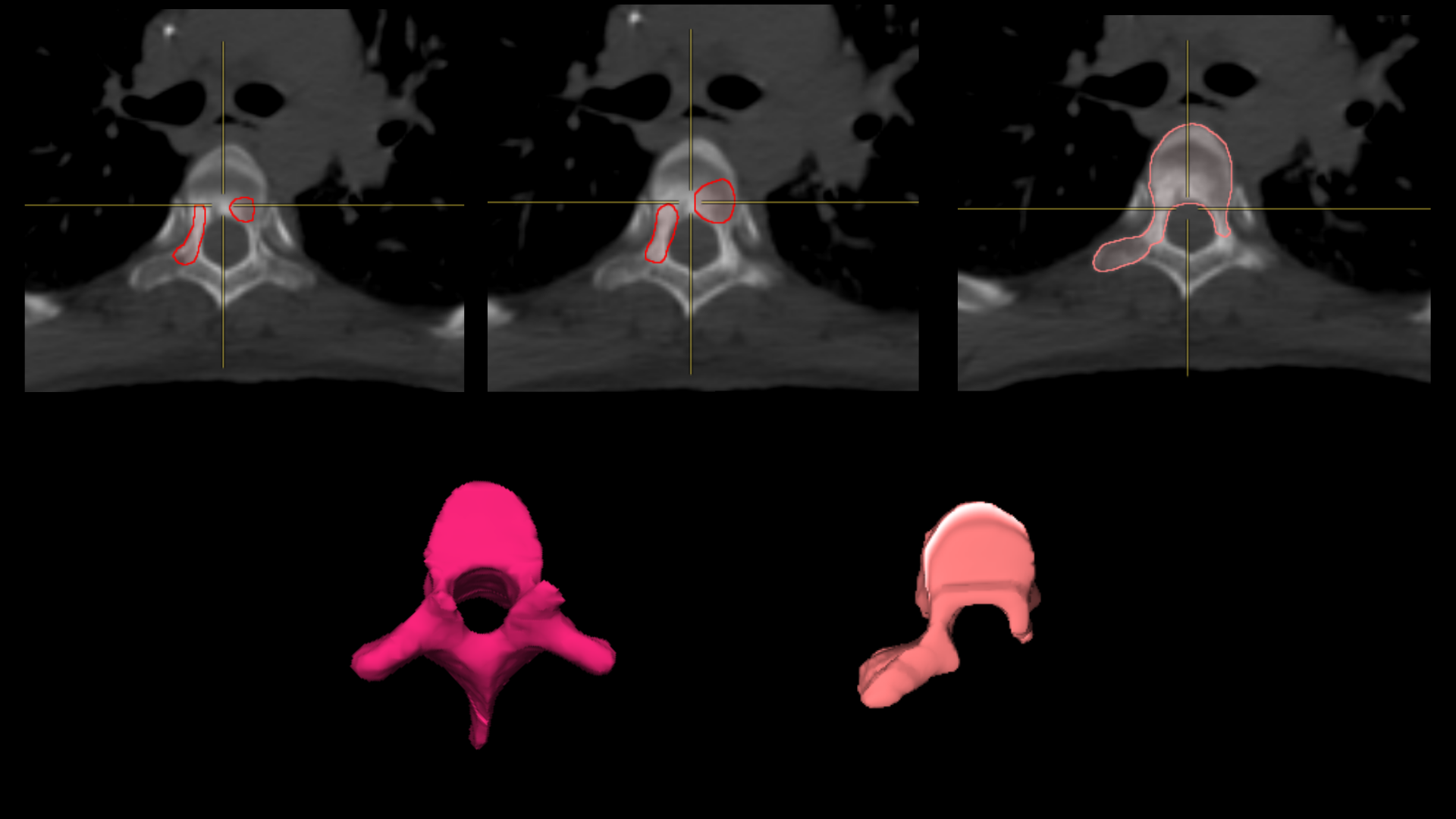

Supplement: Supplementary file 1 [file DataSheet_1.docx]
